# Cross-linking impacts the physical properties of mycelium leather alternatives by targeting hydroxyl groups of polysaccharides and amino groups of proteins

**DOI:** 10.1016/j.heliyon.2024.e36263

**Published:** 2024-08-13

**Authors:** Antonio d’Errico, Michaela Schröpfer, Anke Mondschein, Adil A. Safeer, Marc Baldus, Han A.B. Wösten

**Affiliations:** a Microbiology, Department of Biology, Utrecht University, Padualaan 8, 3584 CH, Utrecht, the Netherlands; b FILK Freiberg Institute gGmbH, Meißner Ring 1-5, D-09599, Freiberg, Germany; c NMR Spectroscopy, Bijvoet Center for Biomolecular Research, Utrecht University, Padualaan 8, 3584 CH, Utrecht, the Netherlands

**Keywords:** Cross-linking, Fungus, Mycelium material, *Schizophyllum commune*, Tanning

## Abstract

Cross-linking, also called tanning, improves mechanical properties of leather and also increases its enzymatic and thermal stability. As a final product, leather has an ultimate tensile strength (σ) of 8–25 MPa and an elongation at break (ε) of >30 %. Mycelium-based materials are a sustainable alternative to leather. Here, the effect of cross-linkers was assessed on mechanical properties of *Schizophyllum commune* mycelium sheets*.* To this end, glutaraldehyde and N-(3-dimethylaminopropyl)-N′-ethylcarbodiimide (EDC) were used as well as extracts of *Ligustrum vulgare* leaves*,* and bark of *Acacia mearnsii* and *Caesalpinia spinosa.* Untanned sheets had a σ of 7.8 MPa and an ε of 15.2 %, while the best overall combination of strength and elasticity was obtained with 0.1 % glutaraldehyde with a σ of 11.1 MPa and an ε of 14.6 %. Cross-linking also increased enzymatic stability and reduced mycelial water absorption but did not result in increased thermal stability. Fourier transform infrared spectroscopy (FTIR), 1D nuclear magnetic resonance spectroscopy (NMR), and amino acid analysis showed that glutaraldehyde bound both protein amino groups and polysaccharide hydroxyl groups by forming Schiff bases and acetals, respectively. Together, synthetic and vegetable cross-linkers can be used to obtain mycelium materials with leather-like tensile strength.

## Highlights

• Synthetic and vegetable cross-linkers improve tensile strength of mycelium sheets.
• Glutaraldehyde improves enzymatic stability of mycelium sheets.
• Glutaraldehyde reduces water absorption of mycelium sheets.
• Glutaraldehyde forms Schiff bases with protein amino groups in mycelium sheets.
• Glutaraldehyde forms acetals with polysaccharide hydroxyl groups in mycelium sheets.

## 1 Introduction

Leather is a strong, flexible, and durable material derived from the chemical processing of animal hides [1,2]. Increasing concerns regarding greenhouse gas emissions from cattle farming, of which leather is a by-product, have led to the search for more sustainable alternatives [[2], [3], [4]]. Mycelium-based materials may be such an alternative [[3], [4], [5]]. These materials can for instance be made by casting filtered mycelium biomass from a liquid fermentation [4,6]. Despite their advantages from a sustainability perspective, mycelium materials show too poor mechanical properties when compared with leather [2,7,8]. Mycelium materials are typically brittle when untreated and display suboptimal tensile strength when plasticized [[9], [10], [11]]. Mechanical performance of animal hides mainly relies on the strength and stability of the collagen network [2]. Tensile strength (8–25 MPa) and elongation at break (>30 %) of leather are 100–1000 fold higher than those of commercially available microorganism-based alternatives, including mycelium materials [2,12].

Collagen fibres of untreated animal hides will eventually collapse onto each other resulting in a stiff sheet, commonly known as parchment [1]. To prevent the collagen polymers to glue together, tanning is used to introduce synthetic cross-links that separate the fibres and stabilise the overall structure [1,13]. Further effects of cross-linking are an increase in hydrothermal stability, higher resistance of collagen to enzymatic degradation and increased tensile strength [1,14].

Nowadays, about 90 % of the leather is tanned with trivalent chromium (Cr^3+^), an environmentally hazardous cross-linker that has led the leather industry to reintroduce tannins of vegetable origin [1,15]. Based on their chemical structure, vegetable tannins can be divided into condensed and hydrolysable tannins, as well as iridoids [16,17]. Condensed tannins are proanthocyanidins constituted by a flavonoid ring structure, which most commonly have a catechol moiety as the B-ring. On the other hand, hydrolysable tannins are polyesters of gallic or hexahydroxydiphenic acid with glucose [16,18]. Hydroxyl groups present in both these classes of polyphenols can form hydrogen bonds between the protonated amino groups and the amide oxygens of collagen polymers. Additionally, quinoid species derived from the oxidation of condensed tannins can form covalent bonds via nucleophilic Michael addition to the primary amines of the lysine residues present in collagen [17,18]. Iridoids are terpene-derived molecules consisting of cyclopentanopyrans bound to a glucose moiety. These glucose residues can be removed by the plant's own enzymes through a ring-opening deglucosidation, turning iridoids into dialdehydes. These dialdehydes can form covalent cross-links with the free lysine residues of collagen under basic pH conditions, a mechanism well studied for glutaraldehyde tanning [[17], [18], [19]]. Because synthetic cross-linkers consist of small defined molecules, their effects on a material are easier to study than with vegetable tannins. For instance, glutaraldehyde is a dialdehyde that reacts with (hydroxy)lysine residues in collagen, forming a Schiff base [20], while N-(3-dimethylaminopropyl)-N′-ethylcarbodiimide (EDC) is a water-soluble carbodiimide that forms amide linkages between carboxyl and amino groups in proteins such as collagen [21].

The use of cross-linking agents employed in leather manufacturing has been implemented to improve the physical stability and performance of fungal materials [3,11,[22], [23], [24], [25]]. The outer part of the hyphae within the mycelium, called the cell wall, can be used for intra- and inter-hyphal cross-linking. The cell wall of the mushroom forming fungus *Schizophyllum commune* consists of a rigid inner core and a mobile outer fraction [26,27]. The former consists of α‐(1,3)‐glucan, β‐(1,3)(1,6)‐glucan, branched mannose, fucan, and β‐(1,4)‐chitin, while the latter comprises β‐(1,3)(1,6)‐glucan, mannan and α‐glucan, as well as protein. Here the impact of cross-linking was studied on the mechanical stability and performance of mycelium sheets of *S. commune* that has been plasticized with 6 % glycerol. Pods of *Caesalpinia spinosa* (Tara) and bark of *Acacia mearnsii* (Mimosa) were used as representatives of hydrolysable and condensed tannins, respectively, while a *Ligustrum vulgare* (Privet) leaf extract represented secoiridoids (a class of iridoids). Glutaraldehyde and EDC were used as synthetic cross-linkers. All cross-linking treatments resulted in improved mechanical performance, resistance to enzymatic degradation and decreased water absorption, while no clear evidence for increased thermal stability was found. Results with glutaraldehyde indicate that covalent interactions are formed with amino acids in protein and with hydroxyl residues of the polysaccharide within the mycelium materials.

## 2 Experimental section

### 2.1 Strain and culture conditions

A piece of mycelium from the periphery of a 7-day-old colony of *S. commune* strain 4-39 (*MAT*A41*MAT*B41, CBS 341.81) was transferred from a −80 °C stock to a 50 mL Greiner tube containing 20 mL *S. commune* minimal medium (SCMM) [28] and cultivated in the dark at 30 °C and 50 rpm for 5 days. The culture was macerated at 18000 rpm in 100 mL SCMM for 30 s using a Waring Blender (Waring Laboratory, Torrington, England, www.waringlab.com). The mycelium homogenate was incubated for 24 h in a 250 mL Erlenmeyer at 30 °C and 200 rpm. This pre-culture was macerated at 18000 rpm for 30 s and aliquots of 1 g wet weight mycelium were used to inoculate 2 L Erlenmeyers containing 1 L SCMM. Cultures were grown for 7 days in the dark at 200 rpm and 30 °C.

### 2.2 Cross-linking of mycelium sheets

Cultures were filtered using cheese cloth and washed with 3 vol demi water. The mycelium was resuspended in 1 L 9 % glycerol (Honeywell, www.honeywell.com), blended for 6 s with an immersion blender (Kenwood kMix Triblade Hand Blender, www.kenwoodworld.com) and incubated at room temperature (RT) for 2 h. Suspensions were filtered through Miracloth® (Merck Millipore, www.merckmillipore.com) and the mycelium was dried at RT on a flat surface between two layers of cellophane (Embalru, www.embalru.nl). The resulting sheets were soaked in 100 mL plant or synthetic cross-linking solution. Glutaraldehyde (25 %) and N-(3-Dimethylaminopropyl)-N′-ethylcarbodiimide (EDC) were acquired from Sigma Aldrich (www.sigmaaldrich.com). Mimosa bark extract (*Acacia mearnsii*) and grinded tara (*Caesalpinia spinosa*) were acquired from SCRD (www.scrd.fr) and Otto Dille (www.otto-dille.de), respectively. Tannins from privet leaves (*Ligustrum vulgare*) were collected, dried, milled and extracted in demi water at 40 °C. The extracts were concentrated on a rotary evaporator and freeze-dried. After overnight incubation with the cross-linkers at 50 rpm at RT, the sheets were washed with excess demi water, plasticized at RT in 100 mL 6 % glycerol for 6 h at 50 rpm, and dried between two layers of cellophane (see above). Each cross-linker was tested on a minimum of three sheets. Controls were subjected to the same treatment, in the absence of cross-linker.

### 2.3 Cross-linking of mycelium powder

Cultures were filtered using cheese cloth and washed with 3 vol demi water. The mycelium was freeze-dried and ground to a powder under liquid nitrogen using mortar and pestle. Part of the powder was further processed to produce water-extracted cell walls (see amino acid analysis) [29]. Aliquots of the mycelium powder (250 mg; particle size approximately 50 μm) were either soaked in 10 mL cross-linking solution using demi water or in pH 3–8 solutions, set with 0.1 M HCl and 0.1 M NaOH. In the former case, increasing concentrations of glutaraldehyde, EDC, *Ligustrum vulgare, Caesalpinia spinosa* and *Acacia mearnsii* were tested. In the case of different pH conditions, glutaraldehyde was added to a final concentration of 1 %. After 1 h incubation at RT, pH was measured, and samples were shaken for 5 h at 30 °C at 60 rpm in a water bath. The cross-linked mycelium was filtered, washed three times with excess of demi water, and air dried on filter paper.

### 2.4 Cross-linking of schizophyllan

Liquid shaken *S. commune* cultures were filtered through Miracloth®. Schizophyllan was isolated from the culture medium by adding ethanol to a final concentration of 65 % (v/v) [30]. After incubation for 24 h at 4 °C, the precipitated schizophyllan was collected by filtration using a Melitta® coffee filter (Melitta, Minden, Germany, www.melitta.com). Residual ethanol in the polysaccharide precipitate was evaporated at RT for 48 h, followed by freeze drying and grinding the polysaccharide under liquid nitrogen with mortar and pestle. Schizophyllan (250 mg) was soaked in 10 mL 1 % glutaraldehyde solution. After 1 h incubation at RT, pH was measured, samples were shaken for 5 h at 30 °C at 60 rpm in a water bath, and the cross-linked glucans were freeze-dried.

### 2.5 Physical and mechanical properties

A minimum of 7 dog-bone shaped samples were cut from each sheet of mycelium. Thickness of each sample was measured at 3 random positions using a Heidenhain MT1281 electronic calliper (Heidenhain, www.heidenhain.com). Mass was measured with an Entris® II analytical balance (Sartorius, www.sartorius.com). Tensile measurements were performed at RT using an Inspekt Solo 2.5 kN machine (Hegewald & Peschke, www.hegewald-peschke.com) with a preload force of 0.5 N, at ramp displacement of 10 mm min^−1^. Ultimate tensile strength (σ) (MPa) was obtained by dividing the maximum force (N) by the area of the cross-section (mm^2^) of each sample. Elongation at break (ε) (%) was calculated as a percentage of the delta of the gauge length before and after the test. Bulk density of samples treated with glutaraldehyde was calculated by dividing the mass of the specimen by its volume. Water absorption of samples treated with glutaraldehyde was determined in conformity with ASTM D570 [31]. It was measured at six different time points over a 24 h interval and defined as weight increase percentage.

### 2.6 Scanning electron microscopy

Mycelium samples were cut into 1 × 1 cm squares with a scalpel, mounted onto a specimen holder, sputtered with a thin layer of gold and imaged using a scanning electron microscope with back scattered electron detector (FEI Quanta 250 FEG-SEM, Thermofisher, www.thermofisher.com) at an accelerating voltage of 10 kV.

### 2.7 ATR-FTIR spectroscopy

Infrared spectra of mycelium samples were recorded in the range of 4000 cm^−1^ to 500 cm^−1^ using a Nicolet™ iS20 FTIR Spectrometer with a diamond crystal (Thermofisher). A minimum of three scans were recorded for each treatment. The influence of pH and cross-linker concentrations were assessed by calculating the relative absorbance between the peak of interest and a peak representing an inert group.

### 2.8 1D solid-state NMR spectroscopy

^13^C-detected dipolar-based cross-polarization (CP; [32]) and scalar-based (INEPT; [33]) 1D experiments, conducted on a 400 MHz NMR system wide bore magnet (Bruker BioSpin, www.bruker.com), were used to probe rigid and mobile components in the cell wall of *S. commune,* respectively. Samples (without dedicated ^13^C isotopic labeling) were packed into a 3.2 mm Magic Angle Spinning (MAS) rotor. A recycle delay of 2 s was used and 4096 scans were recorded for both CP and INEPT 1D experiments with 32.8 ms (2048 points) of direct ^13^C acquisition time using WALTZ16 ^1^H decoupling [34] at 10 kHz for INEPT experiments and 10 ms (626 points) of direct ^13^C acquisition time using SPINAL64 ^1^H decoupling [35] at 96 kHz for CP experiments. The ^13^C offset was set at 80 ppm with a spectral width of 310 ppm. The ^1^H and ^13^C radiofrequency pulse strengths were set at 96.1 kHz and 47.9 kHz, respectively. For the CP experiments, a ramped (70 %) forward cross-polarization from ^1^H to ^13^C with a contact time of 1.0 ms was applied. Chemical shifts in the ^13^C dimension were calibrated using adamantane as an external reference. NMR data were acquired and processed using TopSpin 3.2 (Bruker BioSpin).

### 2.9 Amino acid analysis

Untreated mycelium powder and mycelium powder cross-linked with 1 % glutaraldehyde (10 mg each) were incubated in 6 M HCl and 0.15 % 2-mercaptoethanol at 110 °C for 20 h. Hydrolysates were dried with a vacuum concentrator and dissolved in 0.2 M lithium citrate buffer, pH 2.2. The same procedure was applied to water-extracted cell walls [29]. Amino acid composition was determined via ion-exchange chromatography with post-column derivatization with ninhydrin, using a Biochrom 30+ Amino Acid Analyzer (Biochrom, www.biochrom.co.uk/). Lysine peaks were used to calculate the number of acid-stable bonds formed. Areas under the lysine peaks were normalized using the sum of the areas of all amino acids, and used to calculate the percentage of amino groups involved in cross-linking. The same ratio was calculated for untreated samples and compared. Each experiment was performed in duplicate.

### 2.10 Thermogravimetric analysis

Thermogravimetric analysis (TGA) was performed using a TGA/DSC 3+ Thermal Analysis System (Mettler Toledo). To this end, 7–15 mg mycelium powder was exposed to temperature scans from 25 to 700 °C at a rate of 10 °C min^−1^ under constant nitrogen flow. Each experiment was performed in triplicate.

### 2.11 Enzymatic degradation of the fungal cell wall

Mycelium powder cross-linked with glutaraldehyde was incubated with a 0.1 M glycine solution at 30 °C and 60 rpm for 30 min to quench unbound aldehydes. The powder was washed excessively with water to remove residual tanning agents and freeze dried. Samples of cross-linked or untreated mycelium powder (10 mg) were resuspended in 1 mL home-made hydrolytic enzyme mix from *Trichoderma harzianum* and incubated for 5 h at 30 °C at 50 rpm. After centrifugation, supernatants were carefully removed. Pellets were washed twice with demi water, freeze-dried, and weighed. Residual biomass was expressed as a percentage of the initial biomass. Release of reducing sugars by enzymatic action was quantified colorimetrically, using Dinitrosalicylic Acid Reagent (DNS) and measuring the absorbance at 540 nm in the supernatant [36]. Each experiment was performed in triplicate.

### 2.12 Statistical analysis

Statistical analysis was performed using IBM SPSS Statistics 29.0 (IBM Corporation, New York, USA). Data were analyzed with two-tailed independent sample T-test, One-Way ANOVA followed by a Bonferroni-Holm post-hoc test, Pearson's correlation, and linear regression (p ≤ 0.05). Homoscedasticity was tested with a Levene's test.

## 3 Results

### 3.1 *Mechanical properties of cross-linked mycelium materials*

**Fig. 1:**
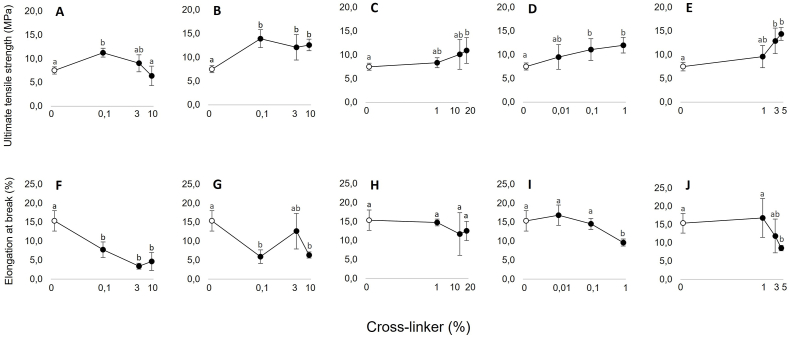
Mechanical properties of mycelial sheets after cross-linking with increasing concentrations of tara (A, F), mimosa (B, G), privet (C, H), glutaraldehyde (D, I) and EDC (E, J). Error bars represent SEM. Different letters indicate significant differences (one-way ANOVA, p < 0.05).

Mycelium sheets of *S. commune* were treated with increasing concentrations of five different cross-linkers, plasticized with 6 % glycerol, and subjected to mechanical testing (Fig. 1). The average ultimate tensile strength (σ) and elongation at break (ε) of the controls were 7.80 MPa and 15.2 %, respectively. A tara concentration of 0.1 % led to an increase of σ (11.3 MPa) and a decrease of ε (7.80 %). An increased tara concentration (3–10 %) resulted in a lower σ (6.4–9.0 MPa), while ε was not affected. Cross-linking with 0.1 % mimosa also resulted in an increased σ (14.0 MPa) but ε was negatively affected (6.0 %). A further increase to 10 % mimosa extract did neither impact σ nor ε. Treatment with privet leaf extract only showed a significant increase in σ at 20 % (the highest concentration used) (10.9 MPa), while it did not impact ε at any of the tested concentrations. In the case of glutaraldehyde and EDC, increasing concentrations (0.01–1 % and 1–5 %, respectively) of the cross-linker led to a nearly proportional increase in σ, while ε only decreased at the highest concentrations. A solution containing 0.1 % glutaraldehyde yielded the best combination of strength and flexibility of all cross-linker treatments with a σ of 11.1 MPa and an ε of 14.6 %. The highest σ was obtained with 5 % EDC (14.4 MPa), while the highest ε was obtained with 0.01 % glutaraldehyde (16.9 %).

### 3.2 Density and water absorption of cross-linked mycelium materials

**Fig. 2:**
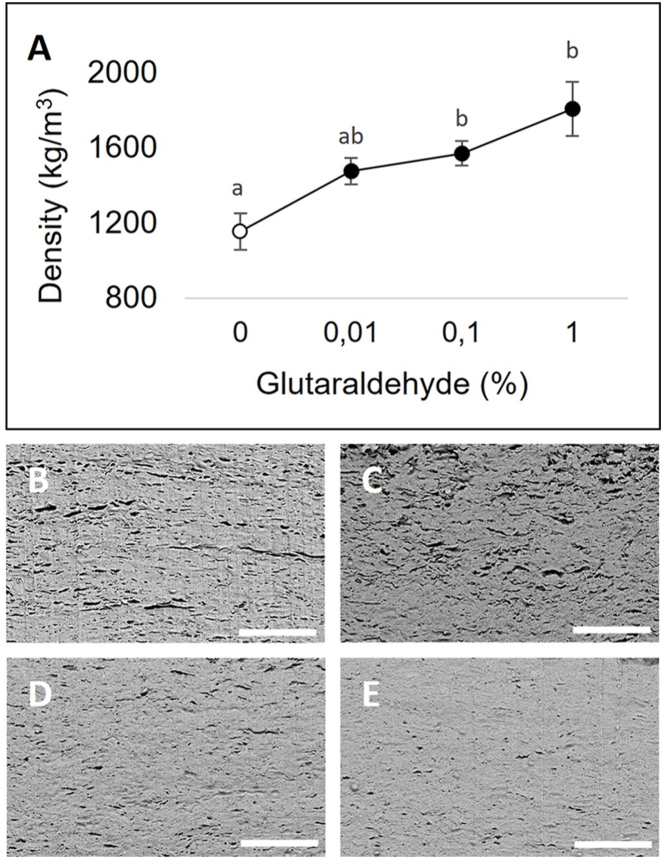
Density of mycelium sheets (A) and scanning electron microscopy of longitudinal sections of mycelium sheets after crosslinking with 0 % (B), 0.01 % (C), 0.1 % (D) and 1 % (E) glutaraldehyde followed by treatment with 6 % glycerol. Increasing the concentration of glutaraldehyde increased the density, which can be explained by the reduced volume of air voids in the sheets. Error bars represent SEM. Different letters indicate significant differences (one-way ANOVA, p < 0.05). White bars represent 20 μm.

Density (ρ) of mycelium materials was determined after crosslinking with 0.01, 0.1 and 1 % glutaraldehyde. Control samples displayed a mean ρ of 1153 kg m^−3^. Treatment with 0.01, 0.1 and 1 % glutaraldehyde increased the density from 1475 to 1569 and 1806 kg m^−3^, respectively (Fig. 2 A). Simple linear regression analysis indicated a relationship between ρ and σ of glutaraldehyde treated mycelium sheets (R^2^ = 0.387; p = 0.03). Scanning electron microscopy revealed that the volume of air voids was increasingly reduced when increasing the amount of cross-linker (Fig. 2B–E).

**Fig. 3:**
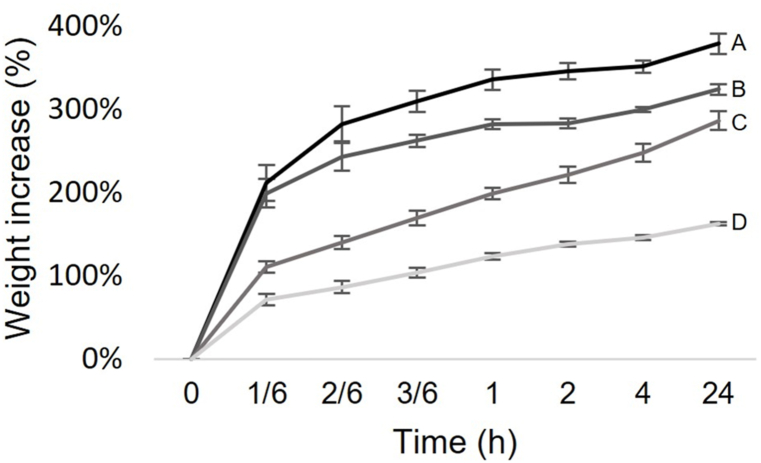
Water absorption of mycelium sheets treated with 0 % (A), 0.01 % (B), 0.1 % (C) and 1 % (D) glutaraldehyde. Mycelium sheets were submerged and weighed at multiple time points over a 24 h period. Error bars represent SEM.

Non-crosslinked glycerol-treated mycelium sheets showed the highest water uptake during a 24 h period (379 %) with most weight increase occurring during the first 10 min of water immersion. Treatment with 0.01, 0.1, and 1 % glutaraldehyde resulted in an increasingly lower water uptake ranging from 324 % to 163 % (Fig. 3).

### 3.3 Enzymatic and thermal stability after cross-linking

**Fig. 4:**
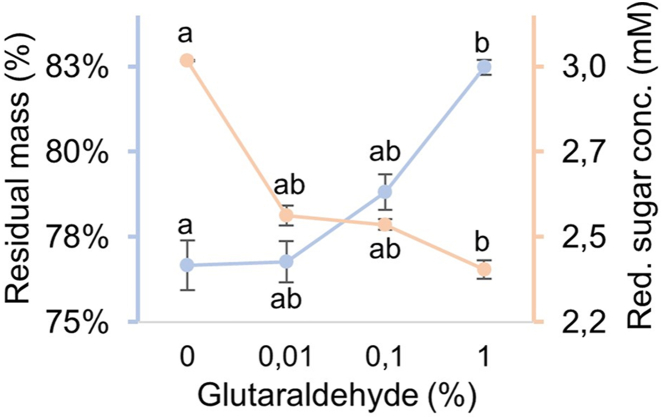
Cross-linking dependent resistance of mycelium to enzymatic degradation expressed as residual mass (%) (blue) and released reducing sugars (mM) quantified via DNS assay (orange). Bars represent SEM. Different letters indicate significant differences (one-way ANOVA, p < 0.05).

Mycelium powder that was either or not cross-linked with 0, 0.01, 0.1, or 1 % glutaraldehyde was treated with lytic enzymes. Non-crosslinked samples showed a mass loss of 23.3 %, while cross-linking with 0.01 or 0.1 % glutaraldehyde did not result in a significant reduction in mass loss when compared to the control. In contrast, 1 % glutaraldehyde treatment did result in resistance to enzymatic degradation with a mean mass loss of 17.5 % (Fig. 4). DNS reducing sugar analysis showed complementary results. Control samples displayed the highest reducing sugar concentration (2.97 mM), indicating higher degrees of enzymatic hydrolysis, while samples treated with 1 % glutaraldehyde resulted in lower sugar release (2.35 mM).

**Fig. 5:**
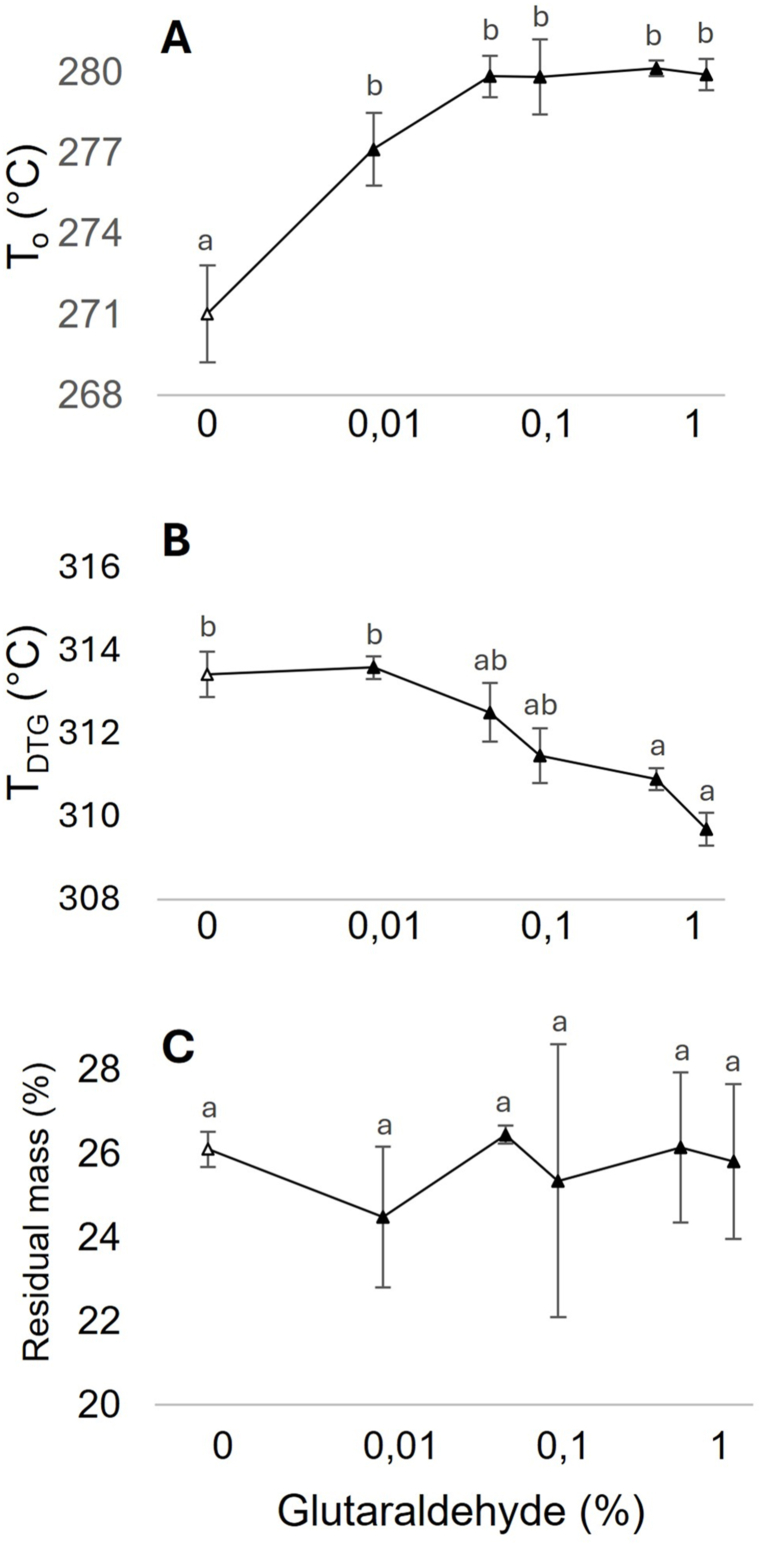
Effect of glutaraldehyde on the thermal stability of mycelium powder. Results from thermogravimetric analysis are expressed as start of decomposition (T_o_) (A), maximum rate of decomposition temperature (T_DTG_) (B) and residual mass percentage (%) (C). Bars represent SEM. Different letters indicate significant differences (one-way ANOVA, p < 0.05).

TGA was used to assess changes in thermal decomposition of mycelium powder after cross-linking with glutaraldehyde (Fig. 5). The onset temperature (T_o_; the start of the material's decomposition) was 271.0 °C in the case of the untreated control, while it ranged between 277.1 and 280.1 °C in the case of 0.01–1 % glutaraldehyde (Fig. 5 A). Conversely, the derivative thermogravimetry peaks of the curve's inflection points (DTG; the temperature at which maximum rate of decomposition occurs) were lower in the case of 0.5 and 1 % glutaraldehyde (310.9 and 319.7 °C, respectively) when compared to the control (313.4 °C) (Fig. 5B). Decomposition residue (expressed as the percentage of the initial sample weight) did not show any significant differences between the samples (Fig. 5C). Together, TGA did not show higher thermal stability after glutaraldehyde cross-linking of mycelium.

### 3.4 Binding mechanism of cross-linking

**Fig. 6:**
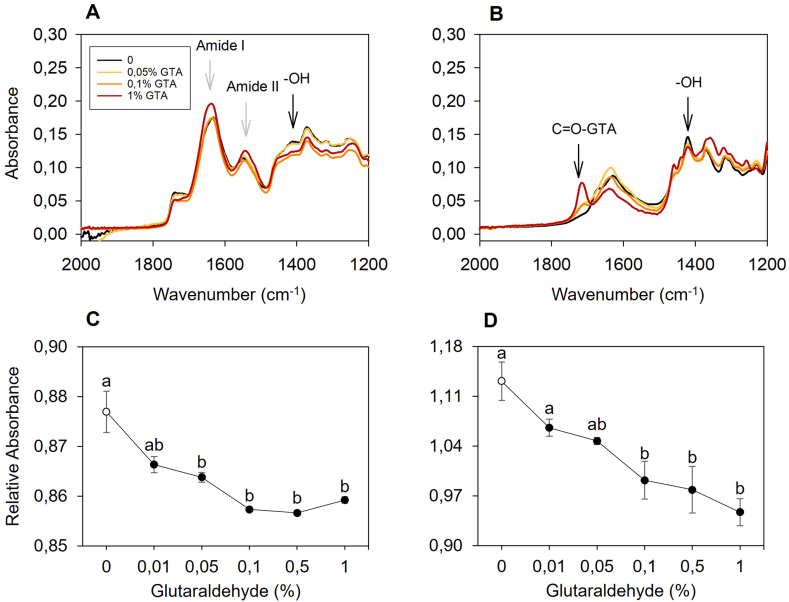
ATR-FTIR spectra of mycelium powder (A) and schizophyllan (B) after treatment with increasing glutaraldehyde concentrations. Black arrow in (A) indicates the drop in hydroxyl signal at wavenumber 1410 cm^−1^. Grey arrows in (A) indicate amide peaks at 1656 cm^−1^ (C=O stretch) and 1546 cm^−1^ (N–H bend). Black arrows in (B) indicate the drop in hydroxyl signal at wavenumber 1410 cm^−1^ and the putative unbound aldehydes at 1720 cm^−1^. Relative absorbance of wavenumber 1410 cm^−1^ to the reference peak at 1375 cm^−1^, showing the progressive decrease in –OH bending for cross-linked mycelium powder (C) and schizophyllan (D). Error bars represent SEM. Different letters indicate significant differences (one-way ANOVA, p < 0.05).

**Fig. 7:**
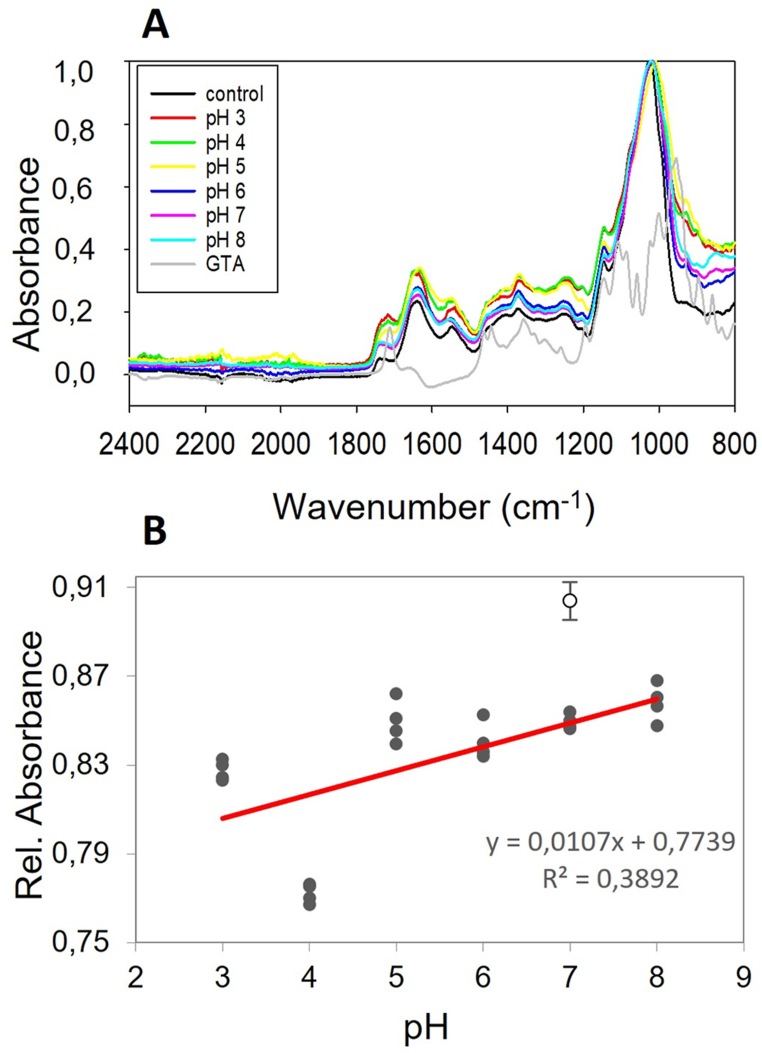
ATR-FTIR spectra of mycelium powder cross-linked with 1 % glutaraldehyde at pH 3–8 (A), and relative absorbance of hydroxyls (1410/1375 cm^−1^) as a function of pH (B). Open circle represents the control, which was only soaked in demineralized water.

ATR-FTIR spectra were collected to elucidate the interaction between mycelium and glutaraldehyde. Changes in peaks, expressed as the relative absorbance to the reference peak at 1375 cm^−1^ (–CH–) were calculated (Fig. 6) [37,38]. Increasing concentrations of cross-linker did not lead to any clear peak decrease at wavenumbers 1656 cm^−1^ (C=O stretch) or 1546 cm^−1^ (N–H bend) (Fig. 6 A), thereby not confirming binding to protein residues. Peaks at wavenumber 1410 cm^−1^ (-OH bend), on the other hand, showed a decrease in relative absorbance at each increase in cross-linker concentration, indicating reactivity of hydroxyl groups to glutaraldehyde (Fig. 6A–C) [38,39]. To remove the noise resulting from the complex composition of mycelium materials, the same experiment was conducted on schizophyllan (β-(1,3)(1,6)-glucan), being a major component of the *S. commune* cell wall [26,27]. Results showed a similar trend, with relative absorbances at wavenumber 1410 cm^−1^ decreasing when the concentration of glutaraldehyde was increased (Fig. 6B–D). An increase in intensity for peaks in the carbonyl region (1720-1740 cm^−1^) of schizophyllan was observed, suggesting the presence of unreacted aldehydes in the samples (Fig. 6 B). No spectral increase could be detected in the carbonyl region of mycelium powder spectra (Fig. 6 A) [40]. The nature of the cross-linking mechanism was further investigated at pH 3–8 using 1 % glutaraldehyde (Fig. 7). Samples cross-linked at all pH values showed a significant drop at wavenumber 1410 cm^−1^ compared to the control, while no significant decrease in relative absorbance was found for Amide I and II regions. A correlation (R = 0.624, p = 0.001) was found between lower pH conditions and the decrease in the relative absorbance at wavenumber 1410 cm^−1^. Simple linear regression (R^2^ = 0.389; p = 0.001) suggests better hydroxyl reactivity to glutaraldehyde at lower pH.

**Fig. 8:**
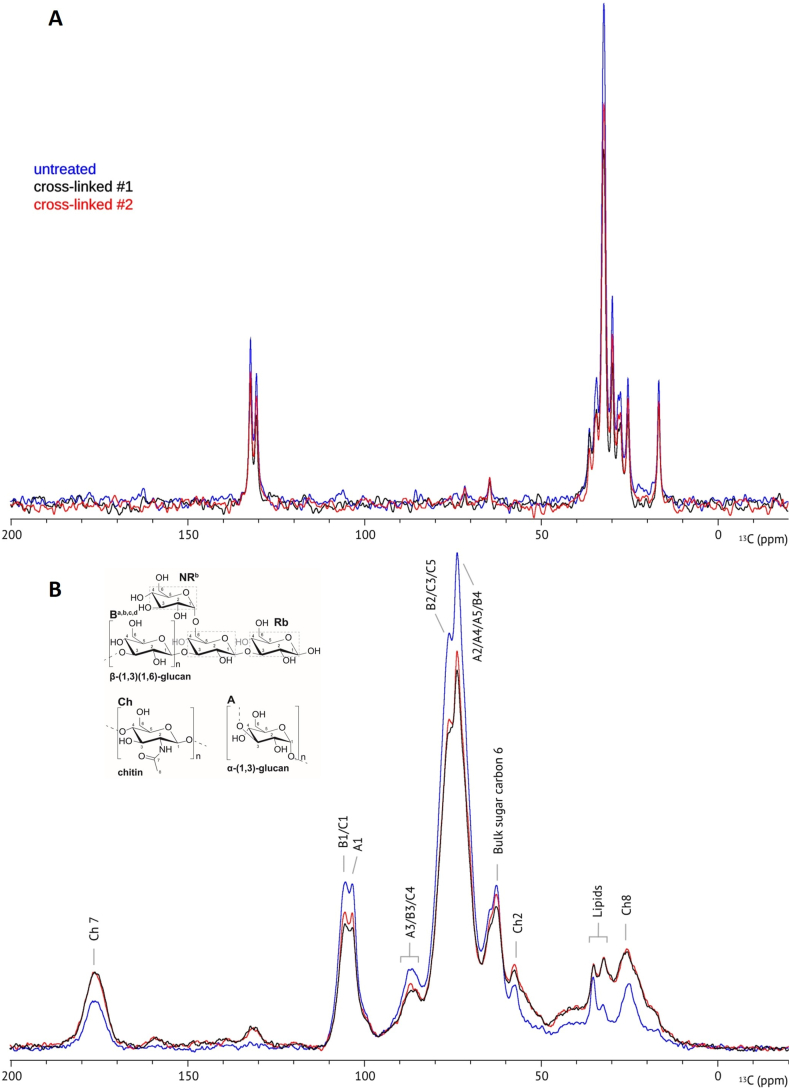
1D solid-state NMR spectra recorded at 400 MHz of the flexible (A) and the rigid (B) part of the cell wall of *S. commune* 4.39 mycelium before (blue) and after cross-linking (red/black) acquired using scalar- and dipolar-based experiments, respectively.

Solid-state 1D NMR spectra were collected from the control and from samples cross-linked with 1 % glutaraldehyde. No significant changes were detected in the flexible part of the cell wall upon glutaraldehyde treatment (Fig. 8 A). In the rigid core, characteristic chemical shifts for chitin were found at 176.2 ppm and at 25.0 ppm, corresponding to the amide carbonyl (C7) and to the methyl group (C8), respectively (Fig. 8 B). Changes in chitin were found after cross-linking, with a significant increase in the relative contribution of C7 and C8. Signal intensity increase after cross-linking was also observed at 32.5 and 35.2 ppm for peaks in the aliphatic region characteristic for lipids, and at 125.0–135.0 ppm, diagnostic for aromatic or alkene resonance. These changes were accompanied by a decrease in intensity at 103.4 and 105.4-ppm, characteristic for anomeric carbon signals of α-linked and β-linked polysaccharides and in the range 66.0–87.0 ppm, diagnostic for glycosidic linkages at C3 and 4 and bulk C6 carbons [26]. Overall, the increased relative contribution of chitin and lipid signals to the spectrum suggest that glutaraldehyde binds to α-linked and β-linked polysaccharides.

**Fig. 9:**
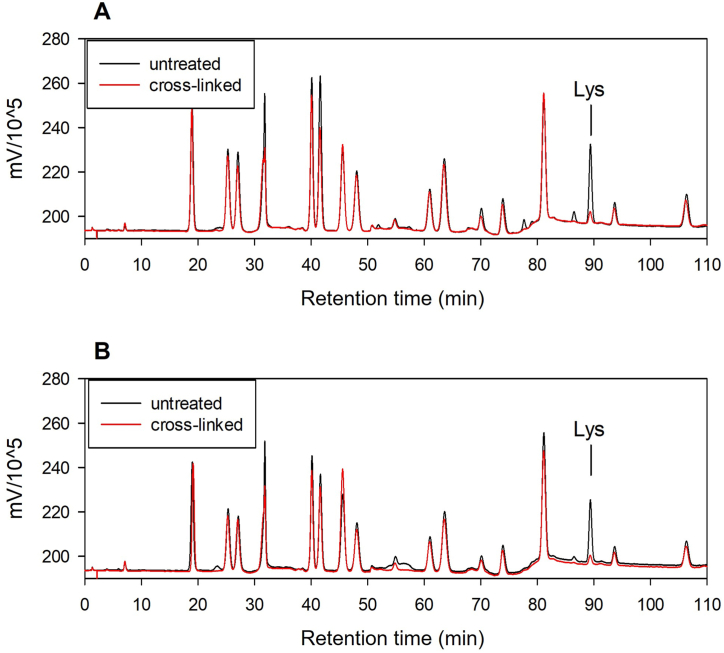
Chromatograms of amino acid composition in untreated and cross-linked mycelium powder (A) and water-extracted cell wall powder (B). Lysine peak is indicated between 88 and 90 min.

Amino acid analysis was performed to investigate the interactions between proteins and cross-linkers (Fig. 9). Total amino acid content, expressed as a percentage of total dry weight was calculated before and after cross-linking. Untreated and cross-linked mycelium powder displayed 16.3 ± 2.23 wt % and 13.5 ± 1.45 wt %, respectively. Untreated and cross-linked water-extracted cell walls consisted of 13.8 ± 0.15 wt % and 11.6 ± 0.10 wt % amino acids, respectively. Lysine comprised 7.00 ± 0.32 wt % and 1.20 ± 0.05 wt % of the total amino acid content in untreated samples and samples cross-linked with 1 % glutaraldehyde, respectively, indicating reactivity between the cross-linker and ε - amino groups of the amino acid (Fig. 9 A). A similar effect was observed in the case of the water-extracted cell wall fraction, with a reduction of lysine residues in the control (7.00 ± 0.05 wt %) to the cross-linked sample (1.30 ± 0.06 wt %) (Fig. 9 B).

## 4 Discussion

Tanning is used to improve the physical, enzymatic and thermal stability of leather. The mechanisms through which cross-linkers react to raw animal hides rely on the substantial presence and availability of protein amino groups and carboxyl groups [18]. It is not clear to what extent proteins are present and available for cross-linking mycelium materials [26,27]. Moreover, the high preponderance of polysaccharides distinguishes mycelium from animal hides. Such structural and chemical differences are likely to induce different binding of cross-linkers. To date, little is known about the effects and the mechanism by which cross-linkers influence mycelium materials. The σ of 19.9 MPa and ε of 2 % of mycelium sheets of *Rhizopus delemar* changed to 7.7 MPa and 5 % and 2.7 MPa and 16 % after treatment with glycerol and glycerol/chestnut wood tannin, respectively (Table 1) [11]. Thus, the chestnut wood tannin reduced strength of the material, while increasing the elasticity. Here opposite effects on the mechanical properties of *S. commune* mycelium sheets were shown using five different cross-linkers.

**Table 1:** Effect of tanning on the mechanical properties of pure mycelium leather alternatives. Results from previous studies are compared with the most effective cross-linker concentrations in the present study.

Fungal species	Treatment	σ (MPa)	ε (%)	Reference
*S.commune*	Untreated	5.0	1.5	[9]
*S.commune*	6 % glycerol	7.8	15.2	
	6 % glycerol + 20 % privet	10.9	12.6	
	6 % glycerol + 0.1 % glutaraldehyde	11.1	14.6	[9]
	6 % glycerol + 0.1 % tara	11.3	7.8	
	6 % glycerol + 0.1 % mimosa	14.0	6.0	
	6 % glycerol + 5 % EDC	14.4	8.6	
*R. delemar*	Untreated	19.9	2	
	20 % glycerol	7.7	5.0	[11]
	20 % glycerol + 1.5 % chestnut	2.7	16.0	
*F. fraxinea*	Hotpress 60 °C	2.7	25.4	
	Hotpress 60 °C + 15 % glycerol	4.9	58.9	[24]
	Hotpress 60 °C + 15 % glycerol + tannic acid	3.1	22.6	

Tanning of mycelium materials enhanced tensile strength for at least one of the concentrations tested for each cross-linking agent. Glycerol-treated mycelium sheets had a σ of 7.8 MPa, while the combination of cross-linking agents and glycerol led to σ values ranging from 10.9 MPa to 14.4 MPa. This was accompanied by an ε of 15.2 % and 6.0–16.9 % in the case of glycerol-treated mycelium and cross-linker/glycerol treated mycelium films. Treatment with 0.1 % glutaraldehyde yielded a material most similar to that of leather with a σ of 11.1 MPa and an ε of 14.6 %. These values are also better than those previously reported using *R. delamar* and *Fomitella fraxinea* (Table 1) [11,24]. It is also shown that plant extracts can be very performative. Use of 20 % *Ligustrum vulgare* (privet) extract resulted in the best properties with σ and ε values of 10.9 MPa and 12.6 %, respectively. The high percentage of *L. vulgare* extract needed to obtain a performative leather-like mycelium sheet is explained by the low amount of tanning molecules. Total polyphenol content in aqueous *L. vulgare* extracts is around 10–20 % (w/w) and consists of flavonoids, phenylpropanoids, hydroxycinnamates, and secoiridoids, while the remaining non-phenolic fraction is largely uncharacterized [41,42]. Secoiridoids, the tanning agents in privet leaves comprise ±3.5 % of the extract [43]. Thus, if completely activated by the plant's glucosidases, the 20 % *L. vulgare* extract would correspond to 0.7 % dialdehyde content, which is in line with the amount of glutaraldehyde that was used in this study.

Glutaraldehyde was used as the model cross-linker to assess additional effects of cross-linking of mycelium material. Cross-linking resulted in an increase in bulk density from 1153 kg m^−3^ in the case of 6 % glycerol-treated mycelium to 1806 kg m^−3^ in the case of 6 % glycerol and 1 % glutaraldehyde treated mycelium. This was explained by the decrease in the volume of air voids but may also be due to the decrease in free volume between molecules [44]. Previously, a correlation was shown between density of the material and the tensile strength [1,45]. However, regression analysis of the data obtained in this study only showed a moderate relationship between ρ and σ. This suggests that other mechanisms are also involved such as decreased mobility of the cross-linked polymer chains [46].

Susceptibility of mycelium to enzymatic action was reduced by 1 % glutaraldehyde, an outcome similar to that after cross-linking of leather [17]. Glutaraldehyde cross-linking also reduced water absorption of mycelium material, which is also in line with previous findings [[47], [48], [49]]. Cross-linking reduces the availability of hydrophilic functional groups, preventing water from binding, as well as the degrees of freedom of the molecules present in the material, forming a physical barrier to the penetration of water [50,51].

Thermal degradation of the components of mycelium (e.g. protein, chitin, and other polysaccharides) has been proposed to occur between 200 and 450 °C [52,53]. Indeed, both T_o_ and DTG were observed to occur within this range for the tested samples. Glutaraldehyde was shown to increase T_o_, while it decreased the DTG. Similar increases in T_o_ have been documented for both glutaraldehyde-cross-linked leather and bacterial cellulose, while tanning dependent reduction of DTG has not been described [[54], [55], [56]]. Together, results do not show higher thermal stability after glutaraldehyde cross-linking of mycelium.

**Fig. 10:**
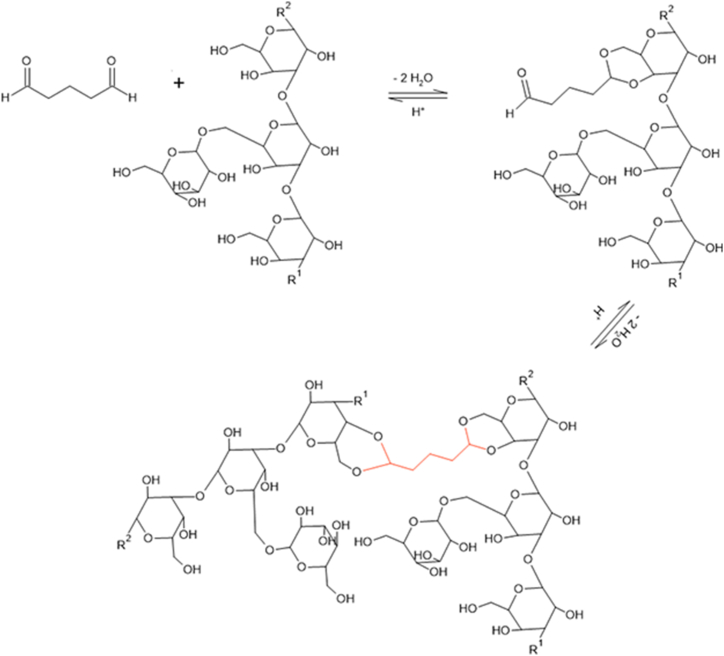
Schematic representation of acetal formation between glutaraldehyde and mycelial β‐(1,3)(1,6)‐glucan. In acidic environment, nucleophilic attack by two hydroxyls of one sugar residue leads to acetalization with one aldehyde (A and B). Through the same reaction another acetal is formed with an adjacent sugar (C). R_1_ and R_2_ represent β-3- and β-1-linked monosaccharide residues, respectively.

Glutaraldehyde is a well-established cross-linker of amine groups within proteins and hydroxyl groups within polysaccharides [20,57]. Amino acid analysis provided evidence for reactivity of L-lysine residues to glutaraldehyde. These proteins may reside in the cell wall since water-extracted cell walls of *S. commune* gave the same results as water washed biomass of this fungus. However, it cannot be excluded that (part of) the proteins reacting with glutaraldehyde originate from the cytoplasm or plasma membrane and have sorbed to the cell wall fraction. In contrast to the amino acid analysis, FTIR and NMR did not provide evidence that glutaraldehyde covalently interacts with proteins within the mycelium material. These spectroscopy techniques did indicate the interaction with hydroxyl groups in polysaccharides. Cross-linking of glutaraldehyde to hydroxyl groups has been reported to take place via acetalization in poly (vinyl alcohol), wood, (methyl) cellulose, and starch [39,45,[58], [59], [60], [61]]. A similar mechanism is proposed to occur in mycelium: the reaction starts with the nucleophilic addition of one hydroxyl in the cell wall polysaccharides to one of the aldehydes, forming a hemiacetal. Subsequently, a second hydroxyl group reacts to form a full acetal. Through the same reaction, the other aldehyde can react to a different portion of the same polysaccharide chain or to a different chain, resulting in a cross-link (Fig. 10) [61]. The moderate relationship that was found between pH and hydroxyl reactivity in cross-linked mycelium supports this hypothesis, as acetal formation optimally occurs under acidic pH conditions [62]. Yet, the formation of new (hemi-) acetals could not be experimentally verified by solid-state NMR. This could be because chemical shifts for most hemiacetal and acetal carbons in polysaccharides will appear between 60 and 110 ppm in ^13^C NMR spectra [63]. Specifically, 1,2-, 1,3-, and 1,4- glycosidic linkages are reported to fall within the 76–87 ppm range, while the anomeric carbon and 1,6- linkages fall in the 66–70 and 90–110 ppm range, respectively [26,63]. The predominant presence of these signals in the spectrum is possibly overlapping with peaks of newly formed acetals or hemiacetals, as these would appear in the same regions.

## 5 Conclusions

Pure mycelium materials are an attractive alternative to leather, but their performance is not yet meeting industry standards. Here it is shown that tanning mycelium leads to improved mechanical properties, lower water absorption and better resistance to degradation by targeting hydroxyls and amino groups. The treated materials demonstrated a tensile strength comparable to that of conventional leather. However, the elongation at break values remain below the optimal range of >30 %. Future investigations should prioritize the optimization of plasticization to improve the material's elasticity. Additionally, the material's tear strength should be evaluated, as this parameter has received insufficient attention in the current body of literature.

## Data availability

Data will be made available by the authors upon request.

## CRediT authorship contribution statement

**Antonio d’Errico:** Writing – review & editing, Writing – original draft, Visualization, Methodology, Investigation, Formal analysis, Conceptualization. **Michaela Schröpfer:** Writing – review & editing, Methodology, Investigation, Conceptualization. **Anke Mondschein:** Writing – review & editing, Funding acquisition, Conceptualization. **Adil A. Safeer:** Writing – review & editing, Methodology, Investigation, Formal analysis. **Marc Baldus:** Writing – review & editing, Supervision, Funding acquisition. **Han A.B. Wösten:** Writing – review & editing, Writing – original draft, Supervision, Funding acquisition, Conceptualization.

## Declaration of competing interest

The authors declare that they have no known competing financial interests or personal relationships that could have appeared to influence the work reported in this paper.
